# Characterization of Monomeric and Dimeric Forms of the Lectin TFF1 in the Human Vagina: Possible Role for the Innate Immune Defence

**DOI:** 10.3390/ijms27062754

**Published:** 2026-03-18

**Authors:** Aikaterini Laskou, Sönke Harder, Eva B. Znalesniak, Hartmut Schlüter, Ines Künnemann, Svetlana N. Tchaikovski, Werner Hoffmann

**Affiliations:** 1 Institute of Molecular Biology and Medicinal Chemistry, Otto-von-Guericke University Magdeburg, Leipziger Str. 44, 39120 Magdeburg, Germany; aikaterini.laskou@klinikum-magdeburg.de (A.L.);; 2 Section Mass Spectrometry and Proteomics, Diagnostic Center, University Medical Center Hamburg-Eppendorf, Martinistr. 52, 20246 Hamburg, Germany; 3 University Clinic for Gynecology and Obstetrics, Otto-von-Guericke University Magdeburg, Gerhart-Hauptmann-Str. 35, 39108 Magdeburg, Germany

**Keywords:** vagina, vaginal microbiota, vaginosis, trefoil factor, TFF, lectin, IgG Fc binding protein, FCGBP, innate immune defence, dermcidin

## Abstract

TFF1 is a secretory polypeptide that is typical of mucous epithelia belonging to the trefoil factor family (TFF) of lectins. Originally, *TFF1* was discovered as an estrogen-responsive gene in breast cancer cell lines. However, its major physiological expression site is the stomach where it exists mainly in a monomeric form, with minor amounts of homodimeric as well as heterodimeric forms, such as a high-molecular-mass complex with IgG Fc binding protein (FCGBP). For the first time, we characterized different low-molecular-mass forms of TFF1 in human post-menopausal vaginal specimens, i.e., monomeric and dimeric forms. Attempts to identify high-molecular-mass forms of TFF1, such as TFF1-FCGBP, failed. Based on its known anti-inflammatory effects, TFF1 could play an important role in the homeostasis of vaginal microbiota, which is normally predominated by *Lactobacillus* spp. Due to its lectin activity, TFF1 might also be capable of binding to members of the vaginal microbiota or to vaginal fungal pathogens. This points to a potential role for TFF1 in the vagina’s innate immune defence and could be of clinical relevance particularly after menopause, e.g., for the treatment of bacterial vaginosis or vulvovaginal candidiasis, as here vaginal dysbiosis is often observed as a consequence of estrogen deficiency.

## 1. Introduction

The vagina is part of the lower female genital tract connecting the outside of the body to the cervix. During prenatal development, the proximal vagina (Müllerian vagina) is epithelialized from the paramesonephric ducts, whereas the epithelium of the distal vagina (sinus vagina) originates from the mesonephric ducts [1,2]. The vagina is covered by a non-keratinized squamous epithelium and contains no glands [3]. The vaginal epithelium of reproductive age women is composed of a layer of basal cells, several layers of parabasal cells, and multiple layers of intermediate and stratified squamous superficial cells, the latter accumulating glycogen [3]. During the menstrual cycle, the migration and stepwise differentiation of basal cells becoming superficial cells is cyclically regulated by estrogen, which results in a periodic thickening of the vaginal mucosa to its maximum at the time of ovulation [3]. At this point in time, superficial cells predominate. Classically, these cytological changes are expressed as a vaginal maturation index (VMI), which represents the ratio of parabasal/intermediate/superficial cells [4]. After the menopause, vaginal atrophy is a common and well-recognized condition due to estrogen deficiency [3]. Thus, in post-menopausal women the vaginal epithelium is thinner and mainly lacks intermediate and superficial cells [5]. Clinically, this atrophy is also characterized by decreased elasticity and lubrication and can be reversed by estrogen treatment, e.g., by topically administered estrogen-containing creams [5], which are able to shift the VMI, e.g., from 94/6/0 to 0/65/35 [6].

The most differentiated superficial cells are known to synthesize and accumulate glycogen under the influence of estrogen. Relative to other epithelial tissues, the glycogen content of vaginal epithelial cells is exceptionally high [7]. At the end of their life cycle, desquamation of superficial cells occurs, and the epithelium replenishes by the mitotic division of cells in the basal layer. After the breakdown of the superficial cells, glycogen is released and used as an energy source, particularly for the bacteria of the *Lactobacillus* genus, which dominate the vaginal microbiota in most women of a reproductive age. The taxonomic composition resembles one of a limited number of configurations termed community state types I, II, III, and V [7,8,9], which are able to metabolize glycogen to lactic acid [7,8,10]. This explains why the vaginal pH in reproductive-aged women is typically 4.5 or less [11,12]. In humans, the vaginal pH is closely linked to estrogen levels and lactobacilli abundance and is lowest when estrogen levels peak just before ovulation [12]. After the menopause, glycogen production decreases, leading to a higher vaginal pH and a shift in the vaginal microbiota, accompanied by an increasing susceptibility to infections [5].

It is worth noting that the human vaginal microbiome is unique among mammals, as *Lactobacillus* spp. comprise >70% of bacteria, compared to <1% in other mammals [12]. This explains why the vaginal pH in non-human mammals is around neutral [11,12]. The reason for this discrepancy might be the high levels of starch in human diets [12].

The predominance of *Lactobacillus* spp. in the human vaginal microbiome, together with the moderately acidic environment, is most strongly associated with reproductive health [9,13]. However, its composition is dynamic and is affected by age, menstrual cycle, ethnicity, lifestyle, and the vaginal mucosal immune system [7,8,9,10,11]. Generally, a depletion of vaginal *Lactobacilli* and dysbiosis of vaginal microbiota is linked with adverse health outcomes, such as preterm delivery, bacterial vaginosis, candidiasis, urinary tract infections, increased risk of sexually transmitted infections, etc. [8,13,14,15,16,17]. It is worth noting that bacteria associated with vaginosis can be suppressed with lactic acid but not hydrogen peroxide [18], probiotics are even beneficial in the prevention of urinary tract infections [19]; a general problem in the treatment of bacterial vaginosis is the formation of biofilms [15,20].

Other than by cervical mucus, the vaginal epithelium is also protected by its own innate immune defence, i.e., by the generation of extracellular reactive oxygen species (ROS) by the NOX/DUOX family of transmembrane NADPH oxidases, such as DUOX1, DUOX2, NOX2, and NOX5, as well as by the secretion of antibacterial lysozymes [21]. The spectrum of defence-related proteins includes even more antimicrobials such as defensins, cathelicidins, secretory leukocyte protease inhibitors (SLPI), elafin, lactoferrin, azurocidin, and dermcidin [22,23]. Furthermore, fucosylation by fucosyltransferase FUT2 probably protects the vaginal epithelium [21] by modulating host–microbe interactions in a manner similar to that of the intestine [24,25]. Another important component is the neonatal Fc receptor (FcRn), which confers protective immunity to vaginal infection [26]. It is worth noting that the binding of FcRn and IgG is markedly dependent on an acidic pH that is typical of the human vagina [26,27].

TFF1 is a secretory polypeptide of the trefoil factor family (TFF) of lectins [28,29,30]. Originally, TFF1 was discovered as an estrogen-responsive gene in a breast cancer cell line [31]. It is worth noting that TFF1 knockdown increased the oncogenic potential of this cell line [32]. However, the major physiological expression sites are surface mucous cells of the gastric mucosa [33]. Mature TFF1 consists of 60 amino acid residues containing seven cysteine residues that form three characteristic intramolecular disulfide bridges in the order Cys^I–V^, Cys^II–IV^, and Cys^III–VI^ [28,34,35]. It is worth noting that these disulfide bonds are unusually stable under reducing conditions and cannot be partially reduced [36]. Cys^VII^ at position 58 is flanked by four glutamic acid residues (EEEC^58^E), and this is probably the reason why TFF1 mainly remains in an unusual monomeric form with a free thiol group, as observed in human, mouse, and *Xenopus laevis* stomachs (ortholog xP1) [37]. These acidic residues are also responsible for binding copper ions [38]. In the stomach, TFF1 is also able to form homodimers and minor amounts of heterodimers with gastrokine 2 (GKN2), IgG Fc binding protein (FCGBP), and an unknown partner protein with a M_r_ of about 50k [37,39,40]. Furthermore, TFF1 is co-expressed with TFF3 and mucins [30] in conjunctival goblet cells [41], efferent tear ducts [42], the false vocal folds of the larynx [42], the nasal mucosa [43,44], the oral mucosa [45], salivary glands [46,47], and the urinary tract [48].

The 5’ flanking region of the *TFF1* gene contains complex enhancer elements responsive to estrogens, epidermal growth factor etc. [49]. Of note, cyclical DNA methylation of the *TFF1* promoter occurs on activation by estrogens [50]. As the gastric mucosa does not express the estrogen receptor, TFF1 expression in the gastric mucosa is estrogen-independent [33].

*Tff1*-deficient (*Tff1*^KO^) mice exhibit mainly a gastric (antral/pyloric adenoma, 30% progressing to carcinomas) as well as an intestinal phenotype (enlarged villi) [51,52,53,54,55]. Nowadays, TFF1 is considered an antral tumour suppressor, possibly regulating Lgr5^+^ antral stem cell differentiation and proliferation [52,56]. Epigenetic silencing of *TFF1* has been observed in gastric cancers of mice and men [57,58] as well as during chronic *Helicobacter pylori* infection [59]. In contrast, in the context of prostate and pancreatic cancers, TFF1 acts as a promoter of tumorigenesis by suppressing oncogene-induced senescence [60].

Homodimeric TFF1 has a lectin activity in vitro recognizing the core oligosaccharide of wild-type *Helicobacter pylori* with an optimum at pH 5.0–6.0 [61,62,63,64,65]. As a consequence, TFF1 induces aggregation and reduces the motility of *H. pylori* [66]. Furthermore, homodimeric TFF1 also binds to gastric mucins from humans, pigs, and *X. laevis* in vitro [64]. In humans, the mucin MUC6 was identified as the target [37]. Generally, N-acetylglucosamine is expected to be a part of the carbohydrate structure recognized by TFF1 [30,64].

TFF1 has been reported to participate in cell differentiation (anti-proliferative and anti-apoptotic effects) [67]. Furthermore, TFF1 seems to negatively regulate inflammatory processes probably via low affinity lectin-mediated binding to and blocking activation of various transmembrane receptors [68,69,70].

The literature concerning TFF1 in the human female reproductive tract is rather sparse. TFF1 expression was detected in trace amounts in the human endometrium [71,72], endocervix [21,73], and vagina [21]. However, TFF1 expression in the vagina is unusual as this epithelium does not synthesize mucins and TFF1 transcripts absolutely predominate when compared with TFF2 and TFF3 transcripts. In contrast, in the endocervix TFF3 transcripts are the predominant TFF transcripts as in most mucous epithelia [21,73]. Thus, it was the aim of this study to investigate the vaginal TFF1 expression of protein levels for the first time and characterize the molecular form(s) of TFF1.

## 2. Results

**Figure 1:**
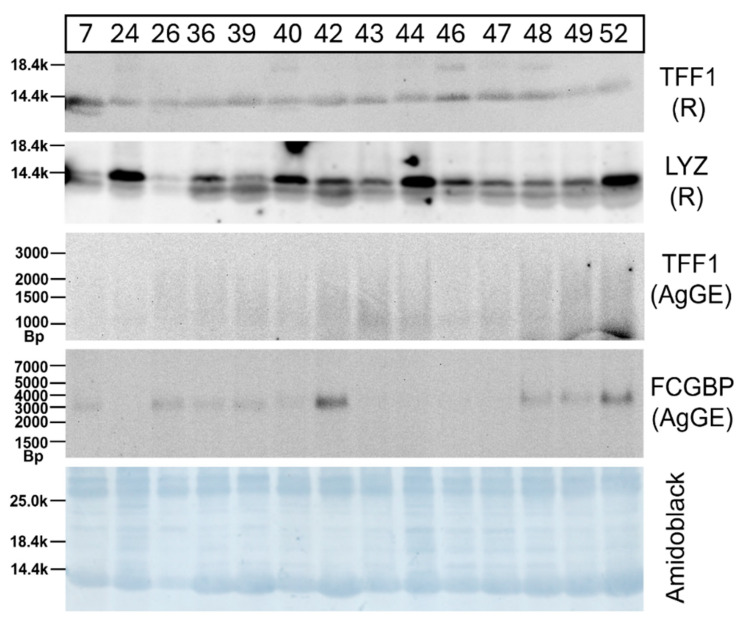
Analyses of 14 human vaginal extracts. Western blot analyses of vaginal extracts (specimens 7, 24, 26, 36, 39, 40, 42–44, 46–49, and 52) concerning TFF1, lysozyme (LYZ) and FCGBP, respectively, are shown after 15% SDS-PAGE under reducing conditions (R; TFF1, LYZ) or 1% AgGE (TFF1, FCGBP). The molecular mass standard is indicated on the left. Relative standard after AgGE: DNA ladder (Bp, base pairs). As a loading control, staining with Amido Black represents the same blot used previously for the detection of LYZ after SDS-PAGE under reducing conditions. Generally, 3 µL (TFF1) or 6.5 µL (LYZ/Amido Black) extract was loaded per lane (SDS-PAGE); for the detection of FCGBP or TFF1 after AgGE, 15 µL extract was loaded per lane.

In the first step, extracts from vaginal specimens were characterized by reducing Western blot analysis for the presence of TFF1 (Figure 1). Furthermore, the content of two secretory products, i.e., low-molecular-mass lysozyme and high-molecular-mass FCGBP, was measured for comparison. Amido Black staining of the Western blot was used as a loading control.

Clearly, TFF1 immunoreactivity was detected in all vaginal samples that were analyzed under reducing conditions (band at about 14k). In contrast, after AgGE, no clear signal was obtained for TFF1 other than a smear in the range between 1000 and 1500 bp. Furthermore, all samples contained lysozyme, which appeared as a double band under reducing conditions. It is worth noting that different patients showed great individual variations in their FCGBP content.

**Figure 2:**
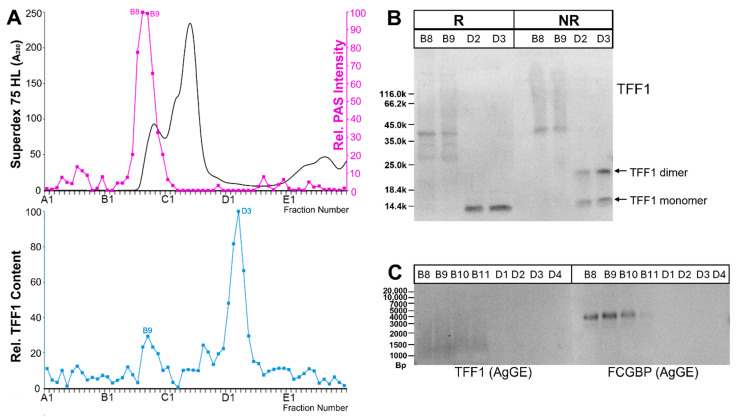
Analysis of human vaginal extract. (**A**) Elution profile of extract V-26 after SEC on a Superdex 75 HL column as determined via absorbance at 280 nm (PAS-positive mucin fractions: pink). Underneath: distribution of the relative TFF1 content as determined by Western blot analysis under reducing conditions and semi-quantitative analysis of monomeric band intensities. (**B**) 15% SDS-PAGE under reducing (R) and non-reducing (NR) conditions, respectively, and Western blot analysis of the high-molecular-mass fractions B8/B9 and the low-molecular-mass fractions D2/D3 concerning TFF1. The molecular mass standard is indicated on the left. (**C**) 1% AgGE and Western blot analysis of the fractions B8–B11 and D1–D4 concerning TFF1 and FCGBP, respectively. Relative standard in (**C**): DNA ladder (Bp, base pairs).

In the second step, extracts from vaginal specimens were separated with the help of size-exclusion chromatography (SEC), and the TFF1 content was measured in each fraction (vaginal specimens V-7, V-24, V-26, and V-36). Generally, low-molecular-mass forms of TFF1 were detectable in all specimens, and there were also faint signals in a single specimen (V-26) in the high-molecular mass range. As a representative example, the results from vaginal specimen V-26 are shown in Figure 2.

The faint TFF1 signal in the high-molecular-mass range under reducing conditions (Figure 2A) turned out to be non-specific (loss of ≤14k band in fractions B8/B9, Figure 2B) in contrast with the TFF1 signal in the low-molecular-mass range (≤14k band in fractions D2/D3, Figure 2B). Under non-reducing conditions, the low-molecular-mass form of TFF1 (fractions D2/D3, Figure 2B) appeared as a double band, i.e., a monomeric form and a form with a M_r_ of about 22k (Figure 2B). Furthermore, after AgGE FCGBP was detectable in the high-molecular-mass fractions B8–B11 but not in the low-molecular-mass fractions D1–D4 (Figure 2C). Notably, TFF1 was not detectable after AgGE, other than a probable non-specific smear in the range between 1000 and 1500 bp (Figure 2C).

**Figure 3:**
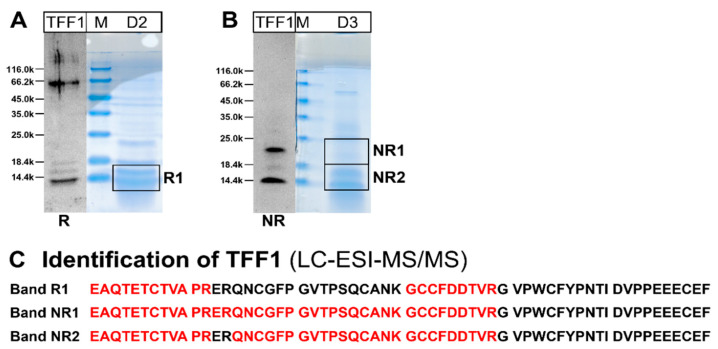
Protein analysis of the low-molecular-mass forms of TFF1 in extract V-26 (fractions D2 and D3, respectively, from Figure 2). (**A**,**B**) Preparative 15% SDS-PAGE under reducing (R) and non-reducing conditions (NR), respectively. Shown are the Western blot analyses concerning TFF1 and parallel Coomassie staining. The molecular mass standard is indicated on the left. Bands R1, NR1, and NR2 were excised. (**C**) Results of the protein analyses after tryptic in-gel digestion of bands R1, NR1, and NR2. Identified peptides in TFF1 are shown in red.

In order to unambiguously verify the TFF1 immunoreactive bands, particularly in the low-molecular-mass region (fractions D2/D3, Figure 2B), the corresponding bands were eluted after reducing and non-reducing SDS-PAGE, respectively (Figure 3A,B), and TFF1 was identified using bottom-up proteomics (Figure 3C). Clearly, the complete N-terminal sequence of mature TFF1 was identified in all three bands as well as internal sequences.

Furthermore, the high-molecular-mass fraction B9 (see Figure 2A,B) was analyzed by the same approach under reducing conditions. However, we were unable to identify TFF1 in that fraction.

**Figure 4:**
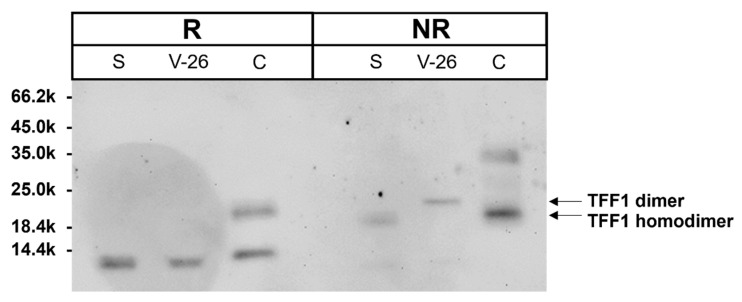
Western blot analysis concerning TFF1 under reducing (R) and non-reducing (NR) conditions: extracts from a human stomach (S), a vagina (V-26) and recombinant human TFF1 (C). The molecular mass standard is indicated on the left.

In the next step, we compared the TFF1 forms in the vagina with the TFF1 forms in the gastric mucosa [37] and recombinant TFF1 [74,75] under reducing and non-reducing conditions (Figure 4).

It is of note that under non-reducing conditions, the dimeric form of vaginal TFF1 appears with a somewhat higher Mr (about 22k) than homodimeric TFF1 from the gastric mucosa and recombinant human TFF1 (both about 19k). This is the first indication that the dimeric form of vaginal TFF1 does not represent a homodimer, but rather a disulfide-linked heterodimer with another partner protein.

## 3. Discussion

Over the course of this study, two low-molecular-mass forms of TFF1 were identified in post-menopausal vaginal extracts, i.e., monomeric and dimeric TFF1 (Figure 2B and Figure 3). The dimeric form differs from that in the gastric mucosa and probably represents a heterodimer with a yet unknown partner protein and a Mr somewhat higher than that of TFF1. A potential candidate could be dermcidin, which is a secretory protein with a Mr of 9.5k and a single cysteine residue that is theoretically capable of forming a disulfide-linked heterodimer with TFF1. It exhibits antimicrobial properties as well as other biological activities [76]. In the past, it has also been characterized in the vaginal fluid [22,76,77], and we were able to identify dermcidin by proteomics in the bands shown in Figure 3. However, all attempts failed to detect dermcidin by Western blot analysis.

Generally, both low-molecular-mass forms of TFF1 are expected to be constituents of the vaginal fluid together with antimicrobial lysozymes. Currently, the molecular function of TFF1 is not understood completely. The motogenic and anti-apoptotic effects of TFF1 are rather weak, arguing against a pronounced role for epithelial restitution [30]. However, the free thiol group of monomeric TFF1 could act as a scavenger for extracellular reactive oxygen/nitrogen species and thus could protect the vaginal epithelium from oxidative damage [30]. Furthermore, TFF1 was reported to interact with various transmembrane receptors, such as IL6Rα-gp80 (and interfering with binding of IL-6), and to suppress the activation of the tumour necrosis factor α receptors (TNFR1, TNFR2) [78,79,80], probably by its lectin activity (lectin-triggered receptor-blocking hypothesis) [56,70]. This might explain the anti-inflammatory effects of TFF1 [68,69,70]. Such an immune modulatory role of TFF1 could well influence the homeostasis of the vaginal microbiota.

Notably, the most apical layers of the vaginal stratified squamous epithelium do not contain classical cell–cell adhesions and are permeable to IgG [81]. Only the suprabasal and basal epithelial layers contain exclusionary junctions, indicating that the uppermost layers of the vaginal epithelium represent a unique microenvironment in host defence against microbial pathogens [81]. Maybe here, due to its lectin activity, TFF1 could act as a defence line against certain members of the vaginal microbiota in a manner comparable to its protective function against *H. pylori* in the stomach [63,66]. Such a lectin-mediated binding of vaginal microbiota could reduce their motility, as was already observed for *H. pylori* [59], and would be supported by the acidic pH typical of the human vagina, as the lectin activity of TFF1 has an optimum pH of 5.0–6.0 [62]. This hypothesis would also explain the preferential expression of TFF1 in epithelia with an acidic luminal pH, such as the stomach and the human vagina. Furthermore, TFF1 could also bind to fungal pathogens, such as *Candida albicans*, and thus could suppress fungal infections in this organ. For example, the lectin Q-Griffithsin suppresses fungal infections in murine models of vaginal candidiasis [82]. Taken together, the lectin TFF1 could play a pivotal role in the human vaginal innate immune defence.

In the future, it would be interesting to test whether TFF1 expression changes during vulvovaginal candidiasis or bacterial vaginosis and whether there is an effect of topically applied estrogen-containing creams on vaginal TFF1 expression. Furthermore, it would be interesting to investigate whether the vaginal TFF1 content changes in pre-menopausal women. Notably, in the endometrium, a cycle phase-specific expression of TFF1 could not be observed [72].

We failed to identify unambiguously high-molecular-mass forms of TFF1 in post-menopausal vaginal extracts, in particular TFF1-FCGBP heterodimers (Figure 1 and Figure 2C). Even a proteomic approach did not detect TFF1 in the high-molecular-mass fraction B9 of extract V-26 (Figure 2A). In the past, the formation of disulfide-linked TFF1-FCGBP heteromers was observed in the human stomach [37] as well as in the murine antrum and duodenum [83]. The reason for a lack of TFF1-FCGBP heteromers in a substantial percentage of human vaginal extracts (Figure 1) might be that FCGBP is hardly synthesized in the post-menopausal vagina [21]. The majority of FCGBP in the vaginal mucus [21] probably results from synthesis in the endocervix, which is a rich source for FCGBP [21]. Thus, FCGBP identified in the vaginal extracts (Figure 1) is probably mostly of endocervical origin, moving downwards into the vagina and is not capable of forming disulfide-linked heterodimers with vaginal TFF1 during the secretory pathway. This might also explain the high individual variations in FCGBP in vaginal extracts observed in Figure 1.

## 4. Materials and Methods

### 4.1. Human Specimens

All investigations followed the declaration of Helsinki and were approved by the Ethics Committee of the Medical Faculty of the Otto-von-Guericke University, Magdeburg (code: 172/21 November 2021). All patients gave written and informed consent. Here, representative results are presented that were obtained with vagina specimens from 14 post-menopausal patients (V-07, V-24, V-26, V-36, V-39, V-40, V-42, V-43, V-44, V-46, V-47, V-48, V-49, and V-52). At least specimens V-07, V-26, and V-42 were obtained from patients who had not received hormone therapy within the last 3 months. Surgical specimens were obtained in the course of resections with a clear clinical indication, e.g., descensus uteri or cystocele and vaginoplasty.

### 4.2. Extraction of Proteins, Protein Purification by SEC

A simplified protein extraction was employed to enable rapid characterization of all vaginal tissue specimens for their TFF1, FCGBP, and lysozyme content. Then, 0.9 g of tissue was homogenized with 1 mL buffer (30 mM NaCl, 20 mM Tris-HCl, pH 7.0, supplemented with protease inhibitors) using a Precellys^®^ 24 lyser/homogenizer (Peqlab Biotechnologie GmbH, Erlangen, Germany) as described [21]. Subsequently, the samples were extracted with chloroform to remove lipids. The upper aqueous phase was centrifuged again to eliminate residual tissue debris, and the resulting supernatant was then subjected to Western blot analysis (Figure 1).

The extraction of vaginal specimens for fractionation by SEC was performed in a similar way with the modification that approximately 1.0 g tissue was minced with a scalpel and distributed to about 10 vials, each extracted with 1 mL buffer (30 mM NaCl, 20 mM Tris-HCl, pH 7.0 plus protease inhibitors) in a Precellys^®^ 24 lyser/homogenizer. After chloroform extraction, 5 mL of the aqueous extracts were fractionated by SEC with the ÄKTA^TM^ FPLC system (Amersham Biosciences, Freiburg, Germany; fraction numbering: A1–A12, B1–B12, etc.) using a HiLoad 16/600 Superdex 75 prep grade column (GE Healthcare Bio-Sciences AB, Uppsala, Sweden); S75HL; 20 mM Tris-HCl, pH 7.0, 30 mM NaCl plus protease inhibitors; flow rate: 1.0 mL/min; 2.0 mL fractions).

### 4.3. SDS-PAGE, AgGE, and Western Blot Analysis

Denaturing SDS-PAGE under reducing and non-reducing conditions, respectively, native AgGE, and Western blot analysis were described previously [21,40]. As a relative standard for non-denaturing AgGE, a DNA ladder was used as specified previously [84].

Human TFF1 was detected with the affinity-purified polyclonal antiserum anti-hTFF1-1 (1:1000 dilution) against the C-terminal peptide FYPNTIDVPPEEECEF of human TFF1 [85]. FCGBP was analyzed with PAP389Hu01 (Cloud-Clone Corp., Katy, TX, USA) against amino acids 5176-5344 of human FCGB. Lysozyme was recognized with a commercial polyclonal antiserum (PA5-16668, Invitrogen by Thermo Fisher Scientific Baltics UAB, Vilnius, Lithuania).

### 4.4. Identification of Proteins by Bottom-Up Proteomics

For protein identification, bands were excised from the gel and subjected to tryptic digestion, followed by liquid chromatography coupled with electrospray-ionization-tandem mass spectrometry (LC-ESI-MS/MS) as reported [84]. The proteomic data were processed and analyzed as described [84].

## 5. Conclusions and Possible Clinical Relevance

Over the course of this study, we identified monomeric and dimeric TFF1 forms in the human post-menopausal vagina. Attempts to identify high-molecular-mass forms of TFF1, such as TFF1-FCGBP, failed. Vaginal TFF1 is expected to play a role for the innate immune barrier. In particular, the reported anti-inflammatory effects of TFF1 [68,69,78,79,80] as well as its known lectin activity [63] possibly also to vaginal microbiota as well as fungal pathogens might regulate their homeostasis and suppress, e.g., fungal infections. This may be particularly important after menopause, as vaginal dysbiosis (e.g., bacterial vaginosis) is often observed as a consequence of estrogen deficiency. Thus, it would be interesting to investigate whether vaginal TFF1 expression is regulated by estrogen as reported in human breast cancer cells [86]. It is worth noting that gastric TFF1 expression is estrogen independent [33].

In the future, it will be absolutely challenging to test whether TFF1 is capable of lectin binding to members of the vaginal microbiota or fungal pathogens, such as *C. albicans*. Furthermore, vaginal application of TFF1 might be beneficial. For example, probiotic delivery of TFF1 by a genetically modified *Lactobacillus lactis* strain would be a possible route. For example, active delivery of TFF1 by recombinant *Lactococcus lactis* as an oral rinse (AG013) has been proven in a phase 1b study in the past to be safe in the treatment of subjects with oral mucositis [87,88,89].

## Data Availability

The original contributions presented in the study are included in the article; further inquiries can be directed to the corresponding author.

## Abbreviations

AgGE	agarose gel electrophoresis
FCGBP	IgG Fc binding protein
LYZ	Lysozyme
PAS	Periodic acid-Schiff
SEC	Size exclusion chromatography
SDS-PAGE	Sodium dodecyl sulfate-polyacrylamide gel electrophoresis
TFF	Trefoil factor family
